# Effect of Freeze Drying, Hot Air Drying, and Hot Air Drying Preceded by Freezing on Phytochemical Composition, Antioxidant Capacity, and Technological Properties of Mango Peels

**DOI:** 10.3390/foods15020333

**Published:** 2026-01-16

**Authors:** Sara Marçal, Ana A. Vilas-Boas, Débora A. Campos, Manuela Pintado

**Affiliations:** Universidade Católica Portuguesa, CBQF—Centro de Biotecnologia e Química Fina—Laboratório Associado, Escola Superior de Biotecnologia, Rua Diogo Botelho 1327, 4169-005 Porto, Portugal; smarcal@ucp.pt (S.M.); avboas@ucp.pt (A.A.V.-B.); dcampos@ucp.pt (D.A.C.)

**Keywords:** antioxidant capacity, carotenoids, drying, free and bound phenolics, fiber, mango byproducts, technological properties

## Abstract

Mango peels have great potential for upcycling in the food industry. This study addressed important knowledge gaps regarding mango peel drying, namely, the effect of drying on mango peels’ bound phenolics, and the impact of prior freezing on the composition of hot air-dried mango peels. Hence, the effect of freeze drying (FD) (0.10 mbar; −63 °C (condenser temperature); 25 °C (shelf temperature); 96 h), hot air drying (HAD) (65 °C; 48 h), and HAD preceded by freezing (FZ + HAD) (−20 °C; 30 days) on mango peels’ composition, antioxidant capacity, and technological properties was evaluated. Drying did not affect fiber content; however, it caused slight modifications in carbohydrate composition of fiber. Regarding antioxidant compounds, FD, HAD, and FZ + HAD reduced vitamin C by 9%, 53%, and 71%, respectively. FD preserved all free phenolics, while HAD and FZ + HAD decreased most of them, with reductions ranging from 20 to 42% and 17 to 71%, respectively. However, FD, HAD, and FZ + HAD reduced 9, 2, and 6 of the 10 bound phenolics identified, respectively, and decreased their antioxidant capacity. Finally, all identified carotenoids were reduced by FZ + HAD, whereas FD and HAD decreased only violaxanthin. Regarding technological properties, FD showed the highest and lowest oil and water absorption capacities. In conclusion, these findings demonstrated that prior freezing exacerbated the loss of antioxidants during HAD.

## 1. Introduction

Mangoes are the sixth most produced fruit worldwide (about 60 million metric tons were produced in 2023) [1]. Mango peels account for approximately 15–20% of the total fruit weight [2]. Typically, whether mangoes are consumed fresh or processed, their peels are rejected due to undesirable sensory characteristics [3]. So, a tremendous amount of mango peels (9–12 million tons) are discarded annually. The deposition of fruit and vegetable byproducts in landfills, or their incineration, causes severe environmental problems, namely greenhouse gas emissions, water and soil pollution, and eutrophication [4,5].

Mango peels have great potential for use in the food industry as a functional ingredient or as a source of natural additives due to their high content of fiber, vitamin C, phenolic compounds (mostly xanthones, flavonoids, and gallic acid and its derivatives), and carotenoids, which ascribe their antioxidant, antimicrobial, anti-diabetic, anti-inflammatory, and prebiotic activities [3,5,6,7].

The valorization of mango peels in the food industry aligns with the Sustainable Development Goals (SDGs) as it offers health, environmental, and economic benefits [8]. In addition to their nutritional value and health benefits, the use of mango peels contributes to solving the environmental and economic problems related to discarding, prevents more natural resources from being spent to produce the ingredients or additives they replace, and can represent a new source of financial income for mango processing companies [9]. However, the valorization of mango peels presents some challenges that need to be overcome, namely their high perishability, seasonality, and variability in chemical composition [8,10,11].

Drying has been widely used to preserve mango peels and other vegetal raw materials [3,6,12,13,14]. Besides extending the shelf life of mango peels by reducing the activity of spoilage microorganisms and enzymes, drying decreases the weight and volume of the peels, facilitating transportation and storage [10,13]. Moreover, processing mango peels into powders makes their incorporation into various types of foods more convenient (e.g., bakery products, pasta, and breakfast cereals) [3,13,14]. However, drying modifies the phytochemical composition, bioactivity, and sensory properties of mango peels. The extent of these changes is strongly affected by drying conditions (temperature, time, process), the kind of pretreatment applied (e.g., blanching, irradiation, pulsed electric field, and pressing), and mango peels’ characteristics (e.g., cultivar, ripeness stage, and postharvest conditions) [15,16,17,18].

A search on Web of Science, in which all papers with the words “drying” in the title/abstract/keywords and “mango peels or mango byproducts/by-products” in any field of the manuscript were analyzed, showed that 22 research studies have been published encompassing the effect of drying on mango peels’ composition and properties since 1991 up to April 2024. Only experimental papers in English that compare fresh mango peels with dried ones, different drying methods, or distinct drying conditions were considered. These 22 research works studied the following drying methods: intermittent microwave–convective drying [19]; drum drying [8,20]; vacuum drying at 60 °C [21]; infrared drying [21]; freeze drying (FD) at −55–−35 °C and 0.01–4.00 mbar [17,18,21,22,23,24,25,26,27]; air drying at room temperature [5]; solar drying [17]; hot air drying in a fluidized-bed at 50–80 °C [15,17,19]; and hot air drying in an oven (HAD) at 45–100 °C without or with constant air circulation (1.50–1.57 m/s) [10,16,17,21,22,23,24,25,26,27,28,29,30,31]. Additionally, some studies evaluated the combination of pretreatments with HAD, namely pulse electric field [32], blanching [18], and washing with ethanol [33]. Finally, Palmeira et al. (2012) did not specify the drying method used [16].

The HAD has been the most studied method for drying mango peels, followed by FD. HAD is simultaneously among the simpler, cheaper, and faster methods, being a methodology that is easily implemented by academia and the food industry [23,25]. Hence, it is a widely adopted technique in academia and the food industry [34]. In its turn, FD is the gold standard for preserving thermolabile compounds [23,25]. The consumer demand for FD fruits and vegetables is increasing. Forecasts indicate that the market value of FD fruits and vegetables will rise to USD 132.7 billion by 2027 [35]. Considering the relevance of these two methods for academia and the food industry, the present study focused on them. After analyzing all papers discussing the effect of FD and HAD on mango peels, important knowledge gaps were identified, and this paper aims to address them.

Although in some research works, the mango peels were frozen before they were submitted to HAD [16,26,27], HAD of fresh mango peels was never compared with HAD preceded by freezing (FZ + HAD). So, the research hypothesis “prior freezing modifies how HAD affects the chemical composition and properties of mango peels” remain unexplored. Freezing can often be necessary as a complementary preserving method to HAD, either in academia or in industry, when mango peels cannot be dried during their shelf life due to, for instance, long transport, limited drying space, and the necessity of evaluating different drying conditions using the same equipment and the same mango peels batch, among others. However, previous studies have shown that freezing leads to tissue damage in mango peels [11,36]. A ruptured food matrix can exacerbate the negative impact of HAD on antioxidant compounds [25].

Moreover, there were no studies about the effect of HAD, FD, and any other drying method on mango peels’ bound phenolic compounds and violaxanthin. Studies with other raw materials have shown that drying conditions strongly affect the bound phenolic content. For instance, Lang et al. (2019) showed that the bound phenolics content in HAD black rice varied depending on drying temperature, while Ma et al. (2023) reported that FD usually converts part of the bound phenolic compounds into free ones [13,37]. Phenolic compounds are in free or bound form, highly influencing their bioaccessibility and, consequently, their health benefits in the human body [38]. Filling this knowledge gap is relevant to upcycling mango peels in the food industry. For instance, the conversion of bound phenolic compounds to free ones could be desired when the final product is a phenolic extract, as it can facilitate extraction [11]. On the other hand, preserving bound phenolics is preferable when mango peels are used as a source of antioxidant fiber [11]. Violaxanthin is a potent peroxidation inhibitor (50 times stronger than β-carotene) [39], so its preservation can be highly desirable when mango peel ingredients/additives are applied in fatty foods as a preservative. Additionally, there are no studies about the impact of FD on mango peels’ vitamin C (one of the main vitamins provided by mango peels), nor the effect of FD and HAD on other polymers from mango peels’ fiber besides the pectin. Fiber is the most important nutrient provided by mango peels, and polymers that compose fibers affect their technological properties and health benefits [3,40].

Considering the knowledge gaps identified above, the present study aimed to evaluate the impact of FD, HAD (65 °C, 48 h), and HAD (65 °C, 48 h) preceded by freezing at −20 °C for 30 days (FZ + HAD) on mango peel powders’ macronutrients, carbohydrate composition of fiber, vitamin C, free and bound phenolic compound profiles, carotenoids, antioxidant capacity, and technological properties. The impact of freezing under the same conditions (−20 °C for 30 days) on the phytochemical composition and antioxidant capacity of mango peels was previously studied by Marçal et al. (2024) [11].

## 2. Materials and Methods

### 2.1. Chemicals

All sugars, phenolic compounds, and carotenoid standards were purchased from Sigma-Aldrich (St. Louis, MO, USA) and Extrasynthese (Genay, France). Trifluoroacetic acid, ascorbic acid, dithiothreitol, phosphoric acid, KH2PO4, 2-azinobis-3-ethylbenzothiazoline-6-sulphonic acid (ABTS), 2,2-azo-bis-(2-methylpropionamidine)-dihydrochloride (AAPH), 2,2-diphenyl-1-picrylhydrazyl (DPPH), Folin–Ciocalteu’s reagent, Trolox, 3,4-dimethylphenol, calcium carbonate, sodium hydroxide, sulfuric acid, hydrochloric acid, and boric acid were acquired at Sigma-Aldrich (St. Louis, MO, USA). Acetone and dichloromethane were obtained from Honeywell Fluka (Morristown, NJ, USA). Hexane and absolute ethanol were purchased from Carlos Erba Reagents (Barcelona, Spain). Methanol was acquired at VWR Chemicals (Radnor, PA, USA). Ethyl acetate and acetonitrile were obtained from Fisher Chemical (Pittsburgh, PA, USA). Christeyns (Agualva—Cacém, Portugal) provided the commercial disinfectant with peracetic acid (Mida Chriox 5).

### 2.2. Raw Materials

All peels analyzed in this study were from the “Tommy Atkins” mangoes (≥12 °bx), cultivated in Brazil, and commercialized in Portugal by the company Nuvi Fruits, SA. Peels used for all assays, except carotenoids and vitamin C, were from mangoes harvested in 2022 and mechanically peeled at Nuvi Fruits, SA. The transportation of these peels from the factory to the laboratory was conducted under refrigerated conditions (ca. 5 °C) within 24 h of the peeling process. Due to equipment constraints, peels used to determine vitamin C and carotenoids profile were harvested in 2024 and manually peeled at the laboratory with a kitchen knife. The manual peeling was carried out to obtain byproducts like those resulting from the mechanical process, ensuring that the amount of mesocarp removed along with the epicarp was similar. Furthermore, after manual peeling, the peels were refrigerated for 24 h to simulate transportation from the factory to the laboratory.

### 2.3. Mango Peels Processing and Storage

First, mango peels were cut by hand with a kitchen knife into roughly 5 g pieces and mixed to ensure similarity between samples submitted to different processing conditions. After, they were washed with peracetic acid following the conditions recommended by Marçal et al. (2022) (mango peels to disinfectant solution ratio: 1:1 (kg:L); peracetic acid concentration: 27 mg/L; disinfection time: 19 min) (fresh sample (FS)) to reduce their organic and microbial load [41].

Then, washed samples were submitted to three different drying processes: FD, HAD, and FZ + HAD. The freeze-dried (FD) samples were frozen at −30 °C and dried with a condenser temperature of −63 °C, a chamber vacuum pressure of 0.10 mbar, and a shelf temperature of 25 °C for 96 h (Gamma 1-16 LSCplus freeze dryer, Martin Christ, Osterode am Harz, Germany). Hot air-dried (HAD) samples were dried at 65 °C for 48 h with a constant airflow of 1.6 m/s (Memmert GmbH + Co.KG, ventilated oven, Schwabach, Germany). FZ + HAD samples were frozen at −20 °C for 30 days and then submitted to the same conditions as HAD mango peels (mango peels were not thawed before HAD). These drying conditions were defined based on the ranges previously reported in the literature (presented in the 6th paragraph of the introduction) and the characteristics of the equipment used.

Finally, the dried peels were milled in a coffee grinder (TAURUS Aromatic II, Barcelona, Spain) for a few seconds until a powder was obtained. Immediately before each assay, fresh peels (about 50 g) were also milled to ensure sample homogeneity and representativeness.

Free phenolic compounds, carotenoids, and vitamin C extractions were performed immediately after processing. Until the remaining assays were performed, mango peel powders were stored in a desiccator at room temperature, while fresh peels were kept at −80 °C. In both, the samples were stored in polyethylene bags. All samples were protected from light throughout storage.

### 2.4. Nutritional Composition

Fresh and dried mango peels were submitted to Standard AOAC (1990) methods [42] to quantify their dry weight (DW) (samples were dried in an oven at 105 °C until constant weight), protein (factor conversion: 6.25) (Kjeldahl method; factor conversion: Nx6.25), and ash (samples were incinerated in a muffle at 550 °C for 6 h). Fat and soluble, insoluble, and total fiber (obtained as the sum of soluble and insoluble fiber) were evaluated following the procedures described by Gómez-García et al. (2021) and Megazyme (2017), respectively [43,44]. Both methodologies are only adequate for dried samples, so these nutrients were determined only in mango peel powders. It was assumed that the fresh peels’ fat, soluble, insoluble, and total fiber were the same as those in the HAD sample. Finally, total carbohydrates were calculated by difference [100 − (% moisture + % ash + % protein + % lipid)].

Regarding data presentation, DW was expressed in g/100 g of sample, while all other nutrients were reported in g/100 g of DW.

### 2.5. Carbohydrate Composition of Soluble and Insoluble Fiber

#### 2.5.1. Hydrolysis

First, the method described by Megazyme (2017) was performed to isolate soluble and insoluble fiber from the other components of mango peel powders [44]. Then, insoluble and soluble fibers were collected from crucibles, crushed, and kept in a desiccator until the hydrolyses were performed.

Two different methodologies, Sluiter et al. (2008) and Ribeiro et al. (2021), were used to hydrolyze insoluble and soluble fiber, respectively [45,46]. Regarding insoluble fiber, first, 250 mg of this nutrient was mixed with 3 mL of sulfuric acid (72% *w:w*) and placed in a water bath at 30 °C for 1 h. Samples were vortexed every 5 min. Afterward, 84 mL of distilled water was added to the samples, and they were autoclaved for 1 h at 121 °C. Finally, autoclaved slurries were filtered through crucibles. The solid particles retained in crucibles were used to quantify acid-insoluble lignin, while filtrates were used to determine neutral sugar profiles and uronic acids [45].

Concerning soluble fiber, first, 30 mg of this nutrient and 12 mL of sulfuric acid (6% *w*:*w*) were mixed using a vortex. Then, these mixtures were autoclaved at 121 °C for 1 h. Finally, the autoclaved solutions were used to determine neutral sugars and uronic acids [46].

#### 2.5.2. Acid-Insoluble Lignin

The crucibles were dried until constant weight at 105 °C before and after filtration, and the amount of ash retained in crucibles along with acid-insoluble lignin was calculated through incineration at 575 °C for 180 min [45]. Hence, the acid-insoluble lignin was calculated by subtracting the weight of ashes from the weight of solid material retained in crucibles. The results were expressed in g/100 g of fiber DW.

#### 2.5.3. Uronic Acids Quantification

Uronic acids were quantified using the colorimetric assay as described by Ribeiro et al. (2020) [47]. Briefly, 250 μL of hydrolyzed fiber or galacturonic acid standards (to perform calibration curve (15–250 μg/mL)) was mixed with 250 μL of a boric acid and chloride acid solution, and 4 mL of sulfuric acid at 96%. Then, they were incubated in a water bath at 70 °C for 40 min. After, 200 μL of dimethylphenol were added to solutions previously cooled at room temperature. Finally, the absorbances of these solutions were read at 400 nm and 450 nm. The absorbances used to create the calibration curve and determine the uronic acid content in the fibers were obtained by subtracting the absorbance at 400 nm from that at 450 nm. The results were presented in g/100 g of fiber DW.

#### 2.5.4. Neutral Sugar Identification and Quantification by HPLC

Neutral sugars were identified and quantified following the method recommended by Sluiter et al. (2008) [45]. First, the pH of solutions with hydrolyzed fiber (5 mL) was adjusted with calcium carbonate powder to values between 5 and 6. Then, the solutions were filtered through a 0.45 µm membrane and analyzed on a HPLC system (Beckman Coulter System Gold HPLC, Knauer, Berlin, Germany) coupled to an infrared detector. The stationary phase was an Aminex HPX-87P Column (300 × 7.8 mm, 9 µm particle size) (Bio-Rad, San Diego, CA, USA) at 85 °C, while the mobile phase was ultra-pure water. The injection volume, flow, and running time were 30 μL, 0.60 mL/min, and 30 min, respectively. Neutral sugars were identified and quantified by comparing peak retention times and areas with calibration curves of pure standards of glucose, xylose, galactose, arabinose, and mannose. The results were shown in g/100 g of fiber DW.

### 2.6. Vitamin C

Brause et al. (2003) described the methodology used to extract and quantify vitamin C in fresh and dried mango peels [48]. Briefly, a solid–liquid aqueous extraction was performed (1 g DW to 40 mL of water) using a dispersing machine (IKA Ultra-turrax T18, Wilmington, DC, USA) at 19,000 rpm for 1 min. Then, the extracts were separated from the residue by centrifugation (3900× *g*, 4 °C, 20 min). Immediately after centrifugation, 1 mg of dithiothreitol was added per mL of extracts, and they were mixed for 2 h in a roller tube at room temperature. Finally, extracts were filtered with 0.22 μm membrane, and vitamin C was quantified by HPLC using the same equipment and methodology described in Marçal et al. (2024) [11]. Identification and quantification of vitamin C were performed by comparing peaks’ retention time, absorption spectrum at 254 nm, and area under a calibration curve of pure standards of ascorbic acid [11,48]. Throughout the process, samples/extracts/standards were always protected from light. The results were expressed in mg/100 g of mango peels DW.

### 2.7. Phenolic Compounds

#### 2.7.1. Methanolic Extraction of Free Phenolic Compounds

Extracts with free phenolic compounds were produced according to Marçal et al. (2022) [41]. First, a solid–liquid methanolic extraction was carried out (1 g of DW to 25 mL of methanol 80%) using a dispersing machine at 24,000 rpm for 1 min. Then, a centrifugation (5000 rpm, 20 min, 4 °C) was performed to separate the extracts containing free phenolic compounds (FPC) from the solid residues (pellet) with bound (non-extractable) phenolic compounds linked.

The extracts (FPC) were stored at −80 °C until they were analyzed, while residues were kept at −20 °C until the hydrolyses of bound phenolic compounds were performed. Both were stored in falcon conical centrifuge test tubes.

#### 2.7.2. Basic and Acid Hydrolyses of Bound Phenolic Compounds

Extracts with bound phenolic compounds were obtained following the procedure described by Marçal et al. (2024) and using the same equipment [11]. Briefly, bound phenolic compounds were released from residues (pellets) of methanolic extraction through a basic hydrolysis with NaOH 4 M for 4 h, at 22 °C and 250 rpm, followed by an acid hydrolysis with HCl 2 M at 85 °C for 1 h. Then, the pH of each hydrolysis supernatant was adjusted to 2, and the supernatants were extracted three times with ethyl acetate. Finally, ethyl acetate was totally evaporated at 30 °C, and dried bound phenolic compounds from basic (BBPC) and acid hydrolyses (ABPC) were dissolved in 2 mL of methanol.

#### 2.7.3. Total Phenolic Compounds

Folin–Ciocalteu method was carried out to quantify total phenolic compounds in the three phenolic extracts of each sample (FPC, BBPC, and ABPC). This methodology was performed following the procedures and using the same equipment described by Vilas-Boas et al. (2020) [49]. Calibration curves were made with gallic acid. Hence, the results were shown in μg of gallic acid equivalents (GAEs)/g of mango peels DW.

#### 2.7.4. Identification and Quantification by HPLC

Identification and quantification of the main free and bound phenolic compounds were performed by HPLC using the same procedure and equipment described in Marçal et al. (2024) [11]. The peaks’ retention time, UV absorption spectrum, and area were compared with calibration curves of pure standards of phenolic compounds. The results were shown in μg/g of mango peels DW.

### 2.8. Carotenoids

#### 2.8.1. Extraction

The extraction of carotenoids was performed according to Stinco et al. (2014) and Marçal et al. (2022) with some modifications [41,50]. Briefly, carotenoids from 2.15 g of fresh mango peels or 0.40 g of mango peel powders were extracted with 5 mL of a hexane/acetone solution (extraction conditions: 1 g of mango peels DW: 12.50 mL of acetone/hexane (1:1; *v*/*v*)), using a dispersing machine for 30 s at 19,000 rpm. After, the slurries were centrifuged for 15 min at 4 °C and 3900 *g* and the supernatants were collected. The extraction was repeated until the residues were colorless. Then, the supernatants from the successive extractions were concentrated using a speed-vacuum evaporator (RVC 2-18, Christ, Osterode am Harz, Germany) for 30 min at 30 °C. Finally, saponification was performed by mixing concentrated extracts with 4 mL of methanolic KOH (30%; *m*/*v*) and 4 mL of dichloromethane in a tube roller for 1 h at room temperature. After removing KOH, the extracts were washed three times with ultra-pure water, and the dichloromethane was evaporated using a nitrogen flow. Dried extracts were resuspended in 1.20 mL of acetone/hexane (1:1; *v*/*v*). Throughout the process, samples/extracts were always protected from light.

#### 2.8.2. Identification and Quantification by HPLC

Carotenoids were identified and quantified by HPLC using the same methodology and equipment as Marçal et al. (2024) [11]. The peaks’ retention time, UV absorption spectrum, and area were compared with calibration curves of pure standards of carotenoids. The results were shown in μg/g of mango peels DW.

### 2.9. Antioxidant Capacity

The capacity of phenolic extracts to scavenge the ABTS and DPPH-free radicals was evaluated following the procedures and using the same equipment described by Vilas-Boas et al. (2020) [49]. Calibration curves were performed with Trolox. Hence, the results were depicted in μg of Trolox equivalents (TEs)/g of mango peels DW.

### 2.10. Technological Properties

Water and oil absorption capacities were evaluated following the methodologies used by Chandra et al. (2015), with slight modifications [51]. First, 1 g of mango peel powders were mixed with 10 mL of deionized water or 10 mL of vegetal oil and kept in a water bath at 60 °C for 30 min. Then, the slurries were centrifuged at 22 °C and 2200 *g* for 15 min. The results were expressed in g of water or oil/g of powders DW.

Regarding the color of mango peel powders, D65 illuminant and the CIE (Commission Internationale de l’Eclairage) parameters (L*, a*, b*) were measured using a colorimeter (Konica Minolta, CR-400, Osaka, Japan). Results were presented as hue angle (h◦ = arctan(b*/a*)).

The particle size distribution of mango peel powders was determined using woven wire mesh sieves (RETSCH, Haan, Germany) and a horizontal sieve shaker (RETSCH, AS 200 Control, Haan, Germany) at an amplitude of 100% for 20 min. The results were presented in g/100 g of mango peel powders.

Water activity (aw) was measured using the LabMaster-aw Neo apparatus (Novasina, Lachen, Switzerland).

### 2.11. Statistical Analysis

Before analyzing and presenting the results, all samples (excluding DW) were converted to 100% DW to offset moisture-related variation.

The statistical analysis was performed with IMB SPSS Statistics Software version 30 (New York, NY, USA). First, the normality of the data distribution was verified using the Shapiro–Wilk test. Then, ANOVA one-way and Turkey’s post hoc tests were carried out (*p* < 0.05).

## 3. Results

### 3.1. Nutritional Composition

Table 1 depicts the nutritional composition of fresh and dried mango peels. Fresh mango peels were mainly composed of water, but as expected, all drying methods reduced the moisture content drastically. Hence, the main constituent of the three mango peel powders was carbohydrates (>90 g/100 g DW). The FZ + HAD sample showed a slight increase in total carbohydrate content (+1%) compared with fresh mango peels and other powders. In all samples, about 37% of total carbohydrates corresponded to dietary fiber. No differences were detected between the soluble, insoluble, and total fiber contents in FD, HAD, and FZ + HAD mango peel powders. The protein content was also similar in all samples. Analysis of fat content showed that the FZ + HAD sample contained 18.11% less fat than the other powders.

Regarding the impact of drying on micronutrients, results showed that FD, HAD, and FZ + HAD did not cause ash reductions; however, they all significantly reduced vitamin C content. FD showed the highest retention of this micronutrient, followed by HAD and FZ + HAD, since they led to a decrease of 8.61%, 53.24%, and 70.52%, respectively, compared with the fresh sample.

### 3.2. Carbohydrate Composition of Soluble and Insoluble Fiber

Table 2 shows the carbohydrate composition of soluble and insoluble fiber from mango peel powders. Uronic acids were the most abundant component in soluble fiber for the three powders, followed by galactose, arabinose, and glucose. No differences were detected between the components of soluble fiber among all samples, excluding glucose, which was higher in FZ + HAD (+46.40% and +90.63% than in FD and HAD, respectively).

Regarding insoluble fiber, the main constituent in the three powders was glucose, followed by acid-insoluble lignin, uronic acids, galactose, xylose, and arabinose. The acid-insoluble lignin content can be slightly overestimated, as discussed in Section 4.1. Insoluble fiber from FD, HAD, and FZ + HAD powders had different contents of glucose and uronic acids, while the other constituents were similar in all samples. The highest glucose and uronic acid contents were detected in FZ + HAD (+7.40% and +16.50% than in HAD and FD) and HAD (+9.18% and +32.24% than in FZ + HAD and FD) samples, respectively.

### 3.3. Phenolic Compounds

Table 3 displays the free and bound phenolic compounds extracted from mango peels by methanolic extraction, and Table 4 shows the bound phenolics released from mango peels by basic and acid hydrolyses. Thirteen phenolic compounds were identified in all samples. Regarding free phenolic compounds, the main ones in FS, FD, and HAD samples were mangiferin and gallic acid, whereas in the FZ + HAD powder, the most abundant were quercetin-3-O-galactoside and gallic acid. The peak identified and quantified as quercetin-3-O-galactoside can also correspond to ellagic acid since they have the same retention time in the HPLC method used. Besides these, six other compounds belonging to the flavonoid and gallate families were also identified as free phenolics.

Regarding bound phenolics, data showed that basic hydrolysis resulted in extracts with a higher diversity of compounds than acid hydrolysis, since in BBPC extracts, ten phenolic compounds were identified, while in ABPC extracts, only four were detected. Gallic acid was the most abundant compound in both extracts (BBPC and ABPC) from all samples, excluding the BBPC extract from FD, since this powder’s main bound phenolic compound was 3,4-dihydroxybenzoic acid. Like in FPC extracts, mangiferin (xanthone) and four different quercetins (flavonoids) were detected in both bound phenolic extracts and only in the BBPC extract, respectively. Furthermore, BBPC and ABPC extracts contained phenolic compounds belonging to the cinnamic acid and other phenolic acid families.

No drying methods affected the diversity of free and bound phenolic compounds. However, they significantly altered the total and individual amounts of these bioactive compounds. Regarding free phenolic compounds, FD showed the highest retention, as it did not reduce the total or any individual phenolic compounds compared with the fresh sample.

Moreover, FD samples presented the highest amount of catechin (+18.60%, +94.86%, and +311.25% compared to FS, HAD, and FZ + HAD samples, respectively). On the other hand, HAD decreased the amount of all free phenolic compounds, excluding penta-O-galloyl-β-D-glucose and gallic acid that were preserved or increased (+35.96% than the fresh sample), respectively. Methyl gallate and catechin were the free phenolic compounds most degraded by HAD (−41.71% and −39.14%, respectively, than in FS), followed by mangiferin (−34.37% than in FS) and the four quercetins identified (c.a. −20% than in FS). Finally, FZ + HAD showed the lowest preservation of free phenolic compounds. Compared with FS, FZ + HAD decreased methyl gallate and catechin by 71.16%, penta-O-galloyl-β-D-glucose by 59.91%, mangiferin by 53.95%, and gallic acid by 16.67%, while the reductions in quercetins, excluding quercetin-3-O-galactoside, were similar to that observed in HAD powders.

The three drying methods decreased the total bound phenolic compounds released through basic and acid hydrolyses. Compared with FS, HAD reduced gallic acid by 71.36% and 74.95% in BBPC and ABPC extracts, respectively, and 4-hydroxybenzoic acid by 76.33% in the ABPC extract. On the other hand, the ferulic acid and 3,4-dihydroxybenzoic acid contents were 40.73% and 71.93% higher in HAD than in FS, respectively, while the other phenolic compounds had similar amounts in both samples. Unlike HAD, comparing with the fresh sample, FZ + HAD reduced quercetin-3-O-galactoside (−27.26%), quercetin 3-O-α-L-arabinopyranoside (−38.49%), p-coumaric acid (BBPC: −15.43%; ABPC: −36.82%), and 3,4-dihydroxybenzoic acid (−51.19%), significantly, and it did not increase ferulic acid. Like HAD, FZ + HAD also decreased gallic acid and 4-hydroxybenzoic acid but, in the last one, FZ + HAD caused reductions in both extracts (BBPC: −30.35%; ABPC: −78%). The amounts of the remaining three bound phenolic compounds (mangiferin, quercetin 3-β-D-glucoside, and quercetin-3-O-α-L-arabinofuranoside) were similar in FS and HAD and FZ + HAD samples.

Finally, FD powder showed the lowest retention of bound phenolic compounds; compared with FS, it exhibited marked reductions in all bound phenolic compounds, except for 3,4-dihydroxybenzoic acid. Furthermore, five phenolic compounds (mangiferin, quercetin-3-O-galactoside, quercetin 3-β-D-glucoside, ferulic acid, 4-hydroxybenzoic acid) showed significantly lower contents in the FD sample than in the other two powders. The lowest reductions were observed in p-coumaric acid (BBPC: −23.40%; ABPC: −31.34%), followed by ferulic acid (−44.89%) and the four quercetins in which the decreases varied between 53.17% and 61.18%, compared with FS. The reductions in the remaining phenolic compounds ranged between 72.31% (mangiferin in BBPC) and 92.00% (gallic acid in BBPC).

### 3.4. Carotenoids

Table 5 presents the carotenoids found in mango peels. β-carotene was the most abundant carotenoid in all samples, followed by lutein and violaxanthin. The peak identified and quantified as lutein can also correspond to zeaxanthin, as discussed in Section 4.2. The three carotenoids showed different susceptibility to drying processes. β-carotene content was unaffected by FD and HAD, while FZ + HAD decreased it by 35.75% compared with the fresh sample. Lutein was the only carotenoid to increase after drying. On the other hand, FD caused no changes in lutein amount, while FZ + HAD reduced it by 22.22%. Violaxanthin was the most susceptible carotenoid to drying processes since its amount in FD and HAD mango peels was 49.47% and 89.89% lower than in fresh samples, respectively, and it was not detected in FZ + HAD powder.

### 3.5. Antioxidant Capacity

Table 6 shows the antioxidant capacity of the free and bound phenolic compounds, measured through ABTS and DPPH assays. Regarding extracts with free phenolic compounds, no differences were detected between FS and the FD sample in both assays. On the other hand, HAD decreased the capacities to scavenge the ABTS and DPPH-free radicals by 43.13% and 48.23%, while FZ + HAD reduced them by 45.03% and 51.79%, respectively, compared with FS.

All drying methods decreased the antioxidant capacity of both extracts with bound phenolic compounds (BBPC and ABPC). Concerning the capacity to scavenge DPPH-free radicals, BBPC and ABPC from FD powders showed the highest reductions (BBPC: −84.94% and ABPC: −79.17%) compared with the FS mango peels. However, HAD (BBPC: −59.35% and ABPC: −48.23%) and FZ + HAD (BBPC: −46.72% and ABPC: −38.20%) also reduced it significantly. Regarding the capacity to scavenge ABTS-free radicals, in BBPC extracts from FD, HAD, and FZ + HAD, the reductions compared with fresh samples were 80.93%, 58.15%, and 50.21%, while in ABPC, they were 74.86%, 57.42%, and 49.33%, respectively. No statistically significant differences between the three powders were found in the ABTS assay.

### 3.6. Technological Properties

Table 7 presents the particle size distribution in the three powders. Excluding the ranges >500 μm and 250–500 μm, where these samples differed by 3.51 and 2.28 g/100 g, respectively, HAD and FZ + HAD powders had a similar particle size distribution. On the other hand, the FD powder had a significantly higher percentage of particles within the smallest ranges measured (50–100 μm and <50 μm) compared with HAD and FZ + HAD samples.

Table 8 displays the technological properties, namely the water and oil absorption capacities, water activity (a_w_), and the color components of the three powders. The three powders had a low water activity. The FD powder showed a lower capacity to absorb water than HAD and FZ + HAD samples (−19.87% and −19.10%, respectively). On the other hand, the FD powder had the highest oil absorption capacity, +45.00% and +58.59%, compared to the HAD and FZ + HAD samples, respectively. No significant differences were detected between HAD and FZ + HAD powders.

Regarding color, L* values showed that the HAD powder was significantly darker than the FD sample, while FZ + HAD powder was the darkest. The three powders’ a* values, b* values, and hue angle were also significantly different. The FD powder presented the lowest a* and b* values and the highest hue angle, followed by HAD and FZ + HAD samples.

## 4. Discussion

### 4.1. Macronutrients and Carbohydrate Composition of Fiber

The values of mango peels’ total carbohydrates, fat, protein, and ash (Section 3.1) agree with data reported previously [3,24]. Regarding the effect of drying, FD and HAD caused no changes in mango peels’ macronutrients, which is corroborated by Garcia-Amezquita et al. (2018) [24]. On the other hand, FZ + HAD reduced total fat, which probably occurred during freezing since Marçal et al. (2024) reported a similar decrease in mango peels submitted to the same freezing conditions [11]. This decrease was likely related to oxidation reactions that break down the fatty acids into smaller volatile compounds that are lost during the fat extraction process [52]. Mango peels are not an important source of fat, so reducing this macronutrient has no relevant impact on their nutritional value.

FD, HAD, and FZ + HAD powders were a great source of fiber since a dose of 100 g DW provided 1.33 times the adequate intake (25 g/day) for this nutrient [53]. Mango peels’ soluble and insoluble fiber vary depending on the cultivar and ripening stage [54]. For instance, Geerkens and Nagel et al. (2015) studied peels from the same mango cultivar and found values very similar to those presented in this study, while Garcia-Amezquita et al. (2018) reported higher values in Ataulfo mango peels [18,24]. Like the present work, Geerkens and Nagel et al. (2015) and Garcia-Amezquita et al. (2018) detected no differences between the fiber content of FD and HAD mango peels [18,24].

In the present study, all polymers that composed the powders’ fiber, excluding lignin, a phenolic polymer, were hydrolyzed in monomeric sugars [45]. The analysis of these simple sugars enabled the deduction of the polymers that composed powders’ soluble and insoluble fiber [40,45,54]. According to previous studies, in insoluble fibers, glucose arose from cellulose, while galactose, xylose, and arabinose came from hemicellulose [40,45,54]. Hence, the results (Section 3.2) suggested that the main polymer in the insoluble fiber of the three powders was cellulose (>30% of insoluble fiber), while the abundance of hemicellulose and acid-insoluble lignin was similar (c.a. 16% of insoluble fiber). The acid-insoluble lignin content may be slightly overestimated due to the presence of acid-insoluble, resistant protein, which was not quantified due to methodological constraints.

In soluble fiber, the main constituent was uronic acids (38–42% of soluble fiber). Uronic acids include galacturonic acids, the main constituent of pectin [40]. Furthermore, galactose and arabinose can also come from branch units of pectin [40]. Glucose was the least abundant sugar in soluble fiber (3–6% of soluble fiber) and possibly came from soluble glucans [54]. Umbreen et al. (2015) reported that cellulose and pectin were the main polymers in mango peel fiber, followed by hemicellulose and lignin, findings that corroborate those of this study [55].

To our knowledge, no previous studies evaluated the impact of FD and HAD on other polymers from mango peels’ fiber besides pectin. Geerkens and Nagel et al. (2015) reported that the yield of alcohol-insoluble solids (composed mainly of pectin) was higher in FD mango peels than in HAD ones [18]. The methodologies used to quantify pectin in the present study and Geerkens and Nagel et al. (2015) were different, which can explain the discrepant findings. FD, HAD, and FZ + HAD powders’ insoluble and soluble fiber differed in glucose content, which suggested that these powders had different cellulose and glucan amounts, respectively. These differences can result directly from disrupting glucans and cellulose during processing or disrupting other polymers that compose fiber, leading to cellulose and glucans concentration [56]. Cellulose is among the most resistant polymers that compose dietary fibers [56]. More studies are needed to clarify it.

Overall, the three drying methods were adequate for fiber preservation. Fiber recovery, namely pectin extraction, represents one of the more usual and economically valuable applications of mango peels in the food industry [3,7].

### 4.2. Antioxidants and Antioxidant Capacity

The major antioxidants in mango peels are vitamin C, phenolic compounds, and carotenoids [3]. This section discusses the effects of FD, HAD, and FZ + HAD on these antioxidants and antioxidant capacity.

#### 4.2.1. Vitamin C

FSs and FD, HAD, and FZ + HAD samples were a great source of vitamin C since a dose of 100 g DW provided 2.80, 2.56, 1.31, and 0.83 times, respectively, the Average Requirements (90 mg/day) [57]. The vitamin C values found in this study were within the range (5–640 mg/100 g DW) reported by Marçal and Pintado (2021) [3]. As observed by Marçal and Pintado (2021), the vitamin C content in mango peels can vary widely [3]. Differences in ripening stage and cultivar, as well as environmental conditions during cultivation, namely light exposure, influence vitamin C synthesis, thereby explaining the variability observed [58].

Although FD showed the highest vitamin C retention, the reduction caused by this drying method was significant (Section 3.1). Several previous studies also reported significant reductions in vitamin C of FD fruits (e.g., mango and pitaya: −28%, papaya −26%, apple: −3%) [13]. Regarding the impact of HAD, Roa-Tort et al. (2024) reported a reduction of 8.14% of vitamin C in HAD (45 °C, 24 h) mango peels compared with fresh ones, while Sogi et al. (2013) found no differences between vitamin C content of HAD (60 °C, 4 h) and FD mango peels [21,28]. In the present study, HAD had more harmful effects on vitamin C content (Section 3.1). Differences in temperatures and drying times can explain these discrepant findings. Dukare et al. (2022) observed that mango peels HAD at 50 °C had a higher vitamin C content than those dried at 70 and 80 °C [10]. Like FZ + HAD, drum drying (130–146 °C; 14–28 s) did not seem to be an adequate alternative method to preserve vitamin C since Troiani et al. (2022) and Antoniolli et al. (2023) reported vitamin C reductions between 61% and 86% in drum-dried mango peels [8,20].

#### 4.2.2. Phenolic Compounds and Antioxidant Capacity

The profiles of mango peels’ free and bound phenolic compounds found in this study agree with data previously reported [3,7,25,59]. For example, Pacheco-Ordaz et al. (2018) and Ancos et al. (2018) also identified mangiferin as the main free phenolic compound [25,59]. Furthermore, Pacheco-Ordaz et al. (2018) also reported a higher diversity of compounds in BBPC extracts than in ABPC extracts and identified gallic acid as the main phenolic compound in both extracts [59]. The higher diversity in BBPC extracts indicated that most mango peels’ bound phenolic compounds were bound to cell walls through ester bonds, since, according to previous studies, basic hydrolysis breaks ester linkages, releasing phenolic compounds from cell walls [59]. On the other hand, acid hydrolysis cleaves glycoside bonds, releasing aglycones [59].

The effect of HAD and FD on mango peels’ total free phenolic compounds has been widely studied [10,21,28,29,32]. These previous works corroborate this study (Section 3.3). For instance, Sogi et al. (2013) reported that the total phenolics content in HAD samples was 27% lower than in FD samples [21]; Dorta et al. (2012) observed a reduction of 53.06% in mango peels submitted to HAD preceded by freezing compared with FD [26]; and Santos et al. (2023) found significant reductions in HAD samples (−44.57% and −57.93% in samples dried at 70 °C and 60 °C, respectively) compared with fresh ones [32].

To our knowledge, no previous studies have simultaneously compared the free phenolic profiles of FS, FD, and HAD mango peels. Nevertheless, Ancos et al. (2018) evaluated this parameter in FD and HAD mango peels, while Berardini et al. (2005) assessed it in FD and HAD mango peels previously frozen at −20 °C [25,27]. Succinctly, Ancos et al. (2018) found that FD mango peels had a higher content of flavonoids and xanthones, while HAD samples had a higher amount of gallic acid and some other gallates, which corroborate the present study (Section 3.3) [25]. Flavonoids and xanthones are more prone to oxidation during HAD due to the high temperature applied and exposure to oxygen [25]. The increases in free catechin and gallic acid in FD and HAD samples (Section 3.3), respectively, can be related to two distinct phenomena, the conversion of some bound phenolics into free ones due to changes in mango peels’ microstructure, as discussed below, and the splitting of complex phenolics into simple ones [13,25,37]. Regarding Berardini et al. (2005), they also reported reductions in mangiferin and some quercetin 3-O-glycosides [27]. However, the magnitude of reductions reported by Berardini et al. (2005) was lower than in the present study, which is probably justified by distinct drying times [27].

Regarding bound phenolics, previous studies have reported two distinct phenomena with opposite effects on these compounds during drying [13,37]. On the one hand, water loss during drying can damage the foods’ microstructure, favoring the release of matrix-bound phenolics and consequently decreasing the amount of bound phenolic compounds [13,37]. In drying methods preceded by freezing, like FD and FZ + HAD, the release of bound phenolic compounds can be heightened since freezing also damages foods’ microstructure [11,13,36]. On the other hand, the drying conditions can favor the complexation of some initial free phenolic compounds with structural constituents increasing bound phenolic compounds [37]. This last phenomenon is a possible explanation for the increase of 3,4-dihydroxybenzoic acid and ferulic acid in BBPC extract from the HAD sample, while the first one justified the drastic reduction of total and some individual bound phenolic compounds, observed in all dried samples compared with FS mango peels.

Additionally, the milling process performed after drying can also contribute to changes in the mango peels’ microstructure and conditioning of the bound phenolic compounds content. Although all samples were submitted to the same milling process before phenolic compound extraction, the resulting powders had different particle size distributions (Section 3.6). The particle sizes of powders also influence the content of free and bound phenolic compounds [60]. Hence, considering that HAD and FZ + HAD had a similar particle size distribution and the data presented in Section 3.3, it can be concluded that HAD had performed better at preserving bound phenolic compounds than FZ + HAD and, consequently, is a better choice when the desired final product is antioxidant fiber. Regarding FD, more studies are needed to clarify its performance compared with HAD and FZ + HAD, since particle size distribution may have acted as a confounding variable.

The present study showed that mango peels had a high antioxidant capacity (Section 3.5) which agrees with the previous literature [3,7,21,26]. As expected, the drying methods that have harmful effects on phenolic compounds also impaired the antioxidant capacity of mango peels [26].

#### 4.2.3. Carotenoids

Like the present study, Ruales et al. (2018) also determined the carotenoids profile of Tommy Atkins mango peels and found the same three carotenoids (β-carotene, lutein, and violaxanthin) [61]. However, in Ruales et al. (2018), the most abundant carotenoid was lutein instead of β-carotene [61]. In its turn, Ancos et al. (2018) corroborated the present study since they identified β-carotene as the main carotenoid in FD and HAD peels from Ataulfo mangoes [25]. Besides the cultivation conditions, other factors like mango cultivar (namely different colored cultivars), postharvest processing, ripening stage, and different epicarp and mesocarp proportions contribute to mango peels’ chemical variability and can explain these discrepancies [11,19,62].

The high susceptibility of violaxanthin to drying, including FD, observed in the present study (Section 3.4) was also reported in other raw materials by previous works [63]. For instance, FD and HAD reduced the violaxanthin by 45.56 and 52.96% in paprika, respectively [63].

The authors found no previous studies that compared the lutein content of FS and FD or HAD mango peels. However, Ancos et al. (2018) compared FD and HAD mango peels and observed that both had the same lutein content [25]. Furthermore, Ancos et al. (2018) did not detect zeaxanthin in the FD sample but found this carotenoid in HAD mango peels [25]. The HPLC method used in the present study to quantify carotenoids did not enable the separation of lutein and zeaxanthin since they had the same retention time. So, the values of lutein presented in Section 3.4 can correspond to the sum of the two compounds, and the increase observed in HAD samples could have resulted from the rise in lutein, zeaxanthin, or both. More studies are needed to clarify the mechanisms that lead to this increase.

Regarding β-carotene, the results of the present study agree with Ancos et al. (2018) since they found no differences between β-carotene content of FD and HAD mango peels [25]. On the other hand, del Pilar Sanchez-Camargo et al. (2019) reported a higher β-carotene content in FD than in HAD mango peels [23]. These discrepant results can be related to differences in mango peels’ microstructure [25]. For instance, Ancos et al. (2018) reported better retention of β-carotene in HAD mango peels than in HAD mango pasta and attributed these discrepant results to differences in the matrix of pasta and peels [25].

Overall, this study showed that FD and HAD were adequate for preserving mango peels’ carotenoids, while FZ + HAD significantly reduced them, so it is inappropriate.

#### 4.2.4. Different Drying Mechanisms and Antioxidants Preservation

FD was better at preserving most antioxidants than HAD and FZ + HAD. Different drying mechanisms justify the distinct performances. In HAD and FZ + HAD, the water was removed by evaporation due to contact between mango peels and hot air [17]. On the other hand, in FD, the water was removed by sublimation under freezing temperatures and low-pressure conditions, which minimized the loss of thermolabile compounds like vitamin C, flavonoids, and xanthones [17]. Furthermore, the exposure of samples to oxygen was lower in FD than in HAD (due to low-pressure conditions versus forced air circulation), which also decreased the deterioration of antioxidant compounds caused by reactions with oxygen [17]. Additionally, during HAD and FZ + HAD, depending on temperatures, enzymes like polyphenol oxidase can remain active until the water activity is lower than 0.6 [17].

FZ + HAD performed worse in preserving antioxidants than HAD. Marçal et al. (2024) submitted mango peels to the same freezing conditions as in the present work, and no significant reductions were detected in vitamin C [11]. Moreover, all individual free phenolic compounds were preserved or increased after freezing, while in carotenoids, only violaxanthin suffered a significant reduction [11]. These previous results indicated that most of the reductions in antioxidants in FZ + HAD samples occurred during the drying step. Marçal et al. (2024) observed that freezing damaged mango peels’ microstructure since the pores became larger or ruptured [11]. These changes in frozen mango peels’ microstructure can justify the bad performance of FZ + HAD regarding the retention of antioxidants since the food structure plays an important role in protecting bioactive compounds from oxidation during drying [25]. In other words, the changes caused by freezing in mango peels’ microstructure make phenolic compounds more extractable but also more susceptible to adverse conditions (e.g., temperature and oxygen) applied during HAD [11,25]. Moreover, damage to the mango peels’ microstructure may have led to greater loss of exudates carrying bioactive compounds during the thawing stage of the drying process. The loss of water and exudates is a common phenomenon during fruit thawing and is frequently associated with reductions in soluble nutrients and bioactive compounds [64]. In FD samples, exposure to high temperatures and oxygen, and the loss of exudates were absent or minimal, since most of the water was removed by sublimation under vacuum and freezing conditions [17]. So, even if prior freezing damaged the microstructure of FD mango peels, this had a much lower impact on the preservation of free phenolics, vitamin C, and carotenoids.

Although FD is the gold standard for preserving antioxidant compounds, it is an environmentally and economically expensive method since a high amount of energy needs to be spent to maintain sublimation conditions [23,24,25]. Moreover, it is a time-consuming method, and the equipment is expensive [23,25]. On the other hand, HAD is a simple, cheap, and fast method. So, even when a mango peel powder with antioxidant properties is the desired final product, HAD could be the most economically and environmentally sustainable method for preserving mango peels. Hence, developing new and sustainable strategies for minimizing losses of mango peels’ antioxidants during HAD is an opportunity for future research. Previous studies with other raw materials showed that pretreatment with natural extracts can be a viable strategy for improving the quality of HAD products [65]. However, to the best of our knowledge, this has not yet been studied in mango peels.

### 4.3. Technological Properties

Factors such as shrinkage, porosity, moisture content of the dried mango peels, and milling equipment characteristics influence the resulting particle size distribution. In the present study, shrinkage and porosity were not measured; however, it is well established that HAD materials generally undergo greater structural shrinkage, whereas FD materials have higher porosity. The higher porosity of FD samples typically reduces their mechanical resistance, facilitating fracture during milling [66]. Geerkens and Nagel et al. (2015) observed that when applying the same milling procedure to FD and HAD alcohol-insoluble solids from mango peels, the first ones resulted in powders with a lower mean particle size, corroborating the present study [18]. The differences in particle size distribution of FD and HAD or FZ + HAD mango peels may have impacted other characteristics of mango peel powders, namely bound phenolics, color, and water and oil absorption capacities [40,60].

The three drying methods reduced the water activity to ≤0.23, thereby extending the shelf life of mango peels by limiting the growth of spoilage microorganisms, as microbial growth is generally inhibited below a water activity of 0.60 [67].The water and oil absorption capacities reported in the present study for FD and HAD mango peel powders were similar to those found by Sogi et al. (2013) [21]. Furthermore, Sogi et al. (2013) also observed that FD powder had a higher capacity to absorb oil, while HAD powder showed a better performance regarding water absorption capacity [21]. The powders’ water and oil absorption capacities are relevant for their application in food products. For instance, powders with high oil absorption capacity help stabilize high-fat products, while powders with high water absorption capacity help prevent syneresis and increase laxative properties [40].

This study evaluated the color components L* (lightness), a* (red-green axis), and b* (yellow-blue axis), and used a* and b* to calculate the hue angle. The reduction in L* values indicates darkening. The darkening of mango peels during drying occurred due to enzymatic browning and Maillard reactions. Moreover, the decrease in the hue angle, as well as the increase in the a* and b* components, can be related to chlorophyll degradation, which led to a reduction in mango peels’ greenness and an increase in their yellowness/redness [10,26]. The results (Table 8 and Section 3.6) suggested that all these deterioration reactions were more intense in FZ + HAD, followed by HAD and FD. As in antioxidant preservation (Section 4.2.2), different drying mechanisms and changes in cell microstructure due to freezing (in FZ + HAD samples) explain these differences. The impact of the darkening of mango peel powders on consumers’ acceptance depends on the characteristics of the foods into which they are incorporated, namely, the color.

## 5. Conclusions

FD, HAD, and FZ + HAD had no statistically significant effect on fiber content. However, they caused slight changes in the fiber’s carbohydrate composition. Regarding antioxidants, the three drying methods impaired (to different extents) the vitamin C, violaxanthin, and bound phenolic compounds content. FD showed the highest retention of vitamin C, free phenolic compounds, and violaxanthin. On the other hand, HAD performed well, preserving carotenoids (excluding violaxanthin) and bound phenolics. The previous freezing exacerbated the loss of antioxidants during HAD, so FZ + HAD was not suitable when the desired product is an antioxidant-rich ingredient/extract or antioxidant fiber. The three drying methods were sustainable for extracting digestible carbohydrates and fiber.

This study evaluated previously unexplored knowledge gaps, specifically examining the impact of freezing as a preliminary step to HAD on the phytochemical composition, antioxidant activity, and technological properties of mango peels. Additionally, it elucidated the effect of FD, HAD, and FZ + HAD on the total and individual bound phenolics and violaxanthin in mango peels. Hence, the present work significantly contributes to the efficiency of upcycling mango peels in the food industry. It provided new information crucial to investigators, food industry professionals, and other stakeholders for interpreting their results and selecting the most appropriate drying conditions for the desired final product. The use of mango peels in the food industry instead of their disposal offers health, environmental, and economic benefits.

## Figures and Tables

**Table 1:** Impact of freeze drying, hot air drying, and hot air drying preceded by freezing on mango peels’ nutritional composition.

	Dry Weight g/100 g	Total Carbohydrates g/100 g DW	Total Fiber g/100 g DW	Soluble Fiber g/100 g DW	Insoluble Fiber g/100 g DW	Protein g/100 g DW	Fat g/100 g DW	Ash g/100 g DW	Vitamin C mg/100 g DW
**FS**	16.83 ± 0.68 ^B^	90.15 ± 0.54 ^B^	33.62 ± 1.43 ^A^*	16.57 ± 0.60 ^A^*	17.69 ± 0.33 ^A^*	5.88 ± 0.74 ^A^	1.92 ± 0.05 ^A^*	2.05 ± 0.34 ^B^	252.41 ± 11.98 ^A^
**FD**	94.37 ± 0.46 ^A^	90.63 ± 0.36 ^AB^	33.31 ± 0.96 ^A^	15.57 ± 0.76 ^A^	17.44 ± 1.10 ^A^	4.96 ± 0.33 ^A^	1.89 ± 0.04 ^A^	2.53 ± 0.00 ^AB^	230.67 ± 3.98 ^B^
**HAD**	90.83 ± 3.27 ^A^	90.24 ± 0.24 ^AB^	33.62 ± 1.43 ^A^*	16.57 ± 0.60 ^A^*	17.69 ± 0.33 ^A^*	4.95 ± 0.08 ^A^	1.92 ± 0.05 ^A^*	2.89 ± 0.20 ^A^	118.02 ± 1.54 ^C^
**FZ + HAD**	93.47 ± 0.78 ^A^	91.26 ± 0.26 ^A^	33.09 ± 1.10 ^A^	16.41 ± 1.06 ^A^	17.69 ± 0.11 ^A^	4.81 ± 0.22 ^A^	1.56 ± 0.02 ^B^	2.36 ± 0.03 ^B^	74.40 ± 1.09 ^D^

Each value was expressed as mean ± standard deviation (n = 3). Different letters in the same column indicate statistically significant differences between samples (*p* < 0.05). * The soluble, insoluble, and total fiber contents and fat amount in fresh mango peels were estimated using the HAD sample since the methodologies applied to quantify these nutrients are inadequate for fresh samples. DW—dry weight; FS—fresh sample; FD—freeze dried sample; HAD—hot air-dried sample (at 65 °C for 48 h); FZ + HAD—samples submitted to freezing (at −20 °C for 30 days) followed by hot air drying (at 65 °C for 48 h).

**Table 2:** Carbohydrate composition of soluble and insoluble fiber from freeze dried, hot air-dried, and frozen and then hot air-dried mango peels.

		Acid-Insoluble Lignin and Protein g/100 g of Fiber	Uronic Acids g/100 g of Fiber	Glucose g/100 of Fiber	Galactose g/100 g of Fiber	Xylose g/100 g of Fiber	Arabinose g/100 g of Fiber
**Soluble Fiber**	**FD**	-	39.87 ± 1.97 ^A^	4.31 ± 0.08 ^B^	21.08 ± 0.82 ^A^	nd	17.58 ± 1.18 ^A^
	**HAD**	-	42.55 ± 1.34 ^A^	3.31 ± 0.05 ^C^	21.11 ± 0.29 ^A^	nd	16.86 ± 0.77 ^A^
	**FZ + HAD**	-	38.22 ± 1.92 ^A^	6.31 ± 0.18 ^A^	20.38 ± 0.78 ^A^	nd	17.47 ± 0.30 ^A^
**Insoluble fiber**	**FD**	16.73 ± 1.17 ^A^	10.70 ± 0.44 ^C^	33.50 ± 0.38 ^C^	8.09 ± 1.33 ^A^	5.83 ± 0.07 ^A^	2.24 ± 0.26 ^A^
	**HAD**	15.72 ± 0.75 ^A^	14.15 ± 0.31 ^A^	36.34 ± 0.41 ^B^	7.30 ± 0.82 ^A^	5.88 ± 0.12 ^A^	2.17 ± 0.54 ^A^
	**FZ + HAD**	15.97 ± 0.76 ^A^	12.96 ± 0.57 ^B^	39.03 ± 0.01 ^A^	6.95 ± 0.49 ^A^	6.34 ± 0.39 ^A^	2.44 ± 0.82 ^A^

Each value was expressed as mean ± standard deviation (n = 3). Different letters in the same column indicate statistically significant differences between samples (*p* < 0.05). FD—freeze dried sample; HAD—hot air-dried sample (at 65 °C for 48 h); FZ + HAD—samples submitted to freezing (at −20 °C for 30 days) followed by hot air drying (at 65 °C for 48 h); nd—not detected.

**Table 3:** Impact of freeze drying, hot air drying, and hot air drying preceded by freezing on free phenolic compounds released from mango peels through methanolic extraction.

	FS μg/g DW	FD μg/g DW	HAD μg/g DW	FZ + HAD μg/g DW
Total phenolic compounds	12,383.18 ± 2147.47 ^A^	10,624.73 ± 1392.74 ^A,B^	7680.63 ± 103.33 ^B,C^	6313.85 ± 333.03 ^C^
Mangiferin	963.77 ± 116.65 ^A^	863.50 ± 10.66 ^A^	632.50 ± 10.11 ^B^	443.81 ± 3.26 ^C^
Gallic acid	610.68 ± 52.39 ^B^	650.32 ± 20.53 ^B^	830.29 ± 9.07 ^A^	508.87 ± 8.96 ^C^
Penta-O-galloyl-β-D-glucose	401.17 ± 50.00 ^A^	384.21 ± 9.76 ^A^	358.72 ± 13.26 ^A^	160.83 ± 50.81 ^B^
Methyl gallate	323.74 ± 53.86 ^A^	299.31 ± 2.22 ^A^	188.72 ± 13.61 ^B^	93.38 ± 5.49 ^C^
Catechin	409.16 ± 19.86 ^B^	485.27 ± 6.62 ^A^	249.03 ± 15.98 ^C^	118.00 ± 14.15 ^D^
Quercetin-3-O-galactoside ^a^	490.95 ± 65.86 ^A^	464.20 ± 3.42 ^A,B^	381.88 ± 0.79 ^B^	513.04 ± 31.56 ^A^
Quercetin 3-β-D-glucoside	183.09 ± 16.69 ^A^	185.26 ± 0.85 ^A^	144.87 ± 0.77 ^B^	138.93 ± 7.56 ^B^
Quercetin 3-O-α-L-arabinopyranoside	79.17 ± 11.46 ^A^	80.08 ± 0.74 ^A^	63.84 ± 0.96 ^B^	53.08 ± 2.02 ^B^
Quercetin-3-O-α-L-arabinofuranoside	52.05 ± 7.71 ^A^	50.59 ± 0.16 ^A^	39.70 ± 0.79 ^B^	35.49 ± 1.54 ^B^

Each value was expressed as mean ± standard deviation (n = 3). Different letters in the same row indicate statistically significant differences between samples (*p* < 0.05). ^a^ In the HPLC method used, quercetin-3-O-galactoside and ellagic acid had the same retention time, so these values can correspond to both these compounds. DW—dry weight; FS—fresh sample; FD—freeze dried sample; HAD—hot air-dried sample (at 65 °C for 48 h); FZ + HAD—samples submitted to freezing (at −20 °C for 30 days) followed by hot air drying (at 65 °C for 48 h).

**Table 4:** Impact of freeze drying, hot air drying, and hot air drying preceded by freezing on bound phenolic compounds released from mango peels through basic and acid hydrolyses, respectively.

	Basic Hydrolysis μg/g DW				Acid Hydrolysis μg/g DW			
	FS	FD	HAD	FZ + HAD	FS	FD	HAD	FZ + HAD
Total phenolic compounds	1075.56 ± 270.66 ^A^	314.40 ± 41.88 ^B^	631.06 ± 46.08 ^B^	644.95 ± 17.84 ^B^	221.95 ± 11.47 ^A^	68.73 ± 11.01 ^C^	113.56 ± 12.62 ^B^	120.91 ± 20.60 ^B^
Mangiferin	34.35 ± 5.84 ^A^	9.51 ± 2.87 ^B^	28.84 ± 6.85 ^A^	22.87 ± 6.61 ^A^	9.21 ± 1.33 ^A^	2.18 ± 0.52 ^B^	6.92 ± 1.50 ^A^	6.25 ± 1.45 ^A^
Gallic acid	533.04 ± 101.22 ^A^	42.65 ± 1.63 ^C^	152.66 ± 26.58 ^BC^	207.74 ± 7.32 ^B^	92.81 ± 25.13 ^A^	12.27 ± 0.27 ^B^	23.25 ± 0.03 ^B^	28.26 ± 1.21 ^B^
Quercetin-3-O-galactoside ^a^	19.99 ± 1.74 ^A^	8.72 ± 1.64 ^C^	18.71 ± 1.72 ^AB^	14.54 ± 3.24 ^B^	nd	nd	nd	nd
Quercetin 3-β-D-glucoside	20.53 ± 4.39 ^A^	7.97 ± 1.80 ^B^	20.38 ± 2.05 ^A^	15.76 ± 0.39 ^A^	nd	nd	nd	nd
Quercetin 3-O-α-L-arabinopyranoside	7.56 ± 1.54 ^A^	3.54 ± 0.01 ^B^	7.71 ± 0.69 ^A^	4.65 ± 1.44 ^B^	nd	nd	nd	nd
Quercetin-3-O-α-L-arabinofuranoside	4.89 ± 1.00 ^A^	2.29 ± 0.36 ^B^	4.71 ± 0.32 ^A^	3.18 ± 0.92 ^AB^	nd	nd	nd	nd
Ferulic acid	6.26 ± 1.32 ^B^	3.45 ± 0.37 ^C^	8.81 ± 0.65 ^A^	6.48 ± 0.08 ^B^	nd	nd	nd	nd
p-coumaric acid	13.29 ± 0.04 ^A^	10.18 ± 0.24 ^B^	14.12 ± 1.27 ^A^	11.24 ± 0.13 ^B^	2.01 ± 0.21 ^A^	1.38 ± 0.15 ^B^	2.04 ± 0.21 ^A^	1.27 ± 0.09 ^B^
3,4-dihydroxybenzoic acid	47.59 ± 3.43 ^B^	52.72 ± 8.84 ^B^	81.82 ± 14.95 ^A^	23.23 ± 0.02 ^C^	nd	nd	nd	nd
4-hydroxybenzoic acid	66.26 ± 9.69 ^A^	16.69 ± 1.20 ^C^	50.39 ± 7.23 ^A,B^	46.15 ± 10.79 ^B^	25.86 ± 1.48 ^A^	2.58 ± 0.25 ^C^	6.12 ± 0.43 ^B^	5.63 ± 0.87 ^B^

Each value was expressed as mean ± standard deviation (n = 3). Different letters in the same row indicate statistically significant differences between samples (*p* < 0.05). ^a^ In the HPLC method used, quercetin-3-O-galactoside and ellagic acid had the same retention time, so these values can correspond to both these compounds. DW—dry weight; nd—not detected; FS—fresh sample; FD—freeze dried sample; HAD—hot air-dried sample (at 65 °C for 48 h); FZ + HAD—samples submitted to freezing (at −20 °C for 30 days) followed by hot air drying (at 65 °C for 48 h).

**Table 5:** Impact of freeze drying, hot air drying, and hot air drying preceded by freezing on mango peels’ carotenoids.

	β-Carotene μg/g DW	Lutein ^a^ μg/g DW	Violaxanthin μg/g DW
**FS**	388.97 ± 11.56 ^A^	15.12 ± 1.14 ^B^	8.47 ± 0.11 ^A^
**FD**	396.77 ± 34.59 ^A^	16.16 ± 1.60 ^AB^	4.28 ± 0.84 ^B^
**HAD**	429.25 ± 20.38 ^A^	18.96 ± 1.46 ^A^	1.11 ± 0.09 ^C^
**FZ + HAD**	249.91 ± 32.64 ^B^	11.76 ± 1.35 ^C^	nd

Each value was expressed as mean ± standard deviation (n = 3). Different letters in the same column indicate statistically significant differences between samples (*p* < 0.05). ^a^ In the HPLC method, lutein and zeaxanthin had the same retention time, so these values can correspond to both these compounds. DW—dry weight; nd—not detected; FS—fresh sample; FD—freeze dried sample; HAD—hot air-dried sample (at 65 °C for 48 h); FZ + HAD—samples submitted to freezing (at −20 °C for 30 days) followed by hot air drying (at 65 °C for 48 h).

**Table 6:** Impact of freeze drying, hot air drying, and hot air drying preceded by freezing on antioxidant capacity of free and bound phenolic compounds released from mango peels through methanolic extraction and basic and acid hydrolyses, respectively.

		ABTS μg of Trolox eq./g DW	DPPH μg of Trolox eq./g DW
**Free phenolic compounds**	**FS**	30,567.99 ± 1940.59 ^A^	28,429.99 ± 3251.63 ^A^
	**FD**	27,262.52 ± 1865.66 ^A^	24,766.32 ± 1729.97 ^A^
	**HAD**	17,384.16 ± 697.05 ^B^	14,717.43 ± 1352.84 ^B^
	**FZ + HAD**	16,804.20 ± 2292.16 ^B^	13,706.52 ± 2313.19 ^B^
**Bound phenolic compounds (basic hydrolysis)**	**FS**	3259.78 ± 809.41 ^A^	2591.03 ± 757.94 ^A^
	**FD**	621.56 ± 28.51 ^B^	390.29 ± 32.31 ^C^
	**HAD**	1364.10 ± 117.82 ^B^	1153.44 ± 109.63 ^BC^
	**FZ + HAD**	1622.91 ± 57.97 ^B^	1380.51 ± 31.00 ^B^
**Bound phenolic compounds (acid hydrolysis)**	**FS**	510.02 ± 92.15 ^A^	346.81 ± 51.87 ^A^
	**FD**	128.20 ± 14.41 ^B^	72.33 ± 14.02 ^C^
	**HAD**	217.18 ± 37.06 ^B^	179.54 ± 8.71 ^B^
	**FZ + HAD**	258.42 ± 55.16 ^B^	214.32 ± 55.81 ^B^

Each value was expressed as mean ± standard deviation (n = 3). Different letters in the same column indicate statistically significant differences between samples (*p* < 0.05). FS—fresh sample; FD—freeze dried sample; HAD—hot air-dried sample (at 65 °C for 48 h); FZ + HAD—samples submitted to freezing (at −20 °C for 30 days) followed by hot air drying (at 65 °C for 48 h).

**Table 7:** Particle size distribution in freeze dried, hot air-dried, and frozen and then hot air-dried samples.

	>500 μm g/100 g	250–500 μm g/100 g	150–250 μm g/100 g	100–150 μm g/100 g	50–100 μm g/100 g	<50 μm g/100 g
**FD**	6.03 ± 0.29 ^C^	11.79 ± 0.16 ^C^	13.92 ± 0.97 ^B^	12.62 ± 1.20 ^A^	31.15 ± 4.95 ^A^	24.49 ± 4.33 ^A^
**HAD**	18.26 ± 1.28 ^A^	26.88 ± 0.96 ^B^	17.22 ± 1.06 ^A^	10.90 ± 0.45 ^A^	15.70 ± 1.58 ^B^	11.03 ± 1.61 ^B^
**FZ + HAD**	14.75 ± 0.41 ^B^	29.16 ± 0.67 ^A^	18.71 ± 0.07 ^A^	12.48 ± 0.48 ^A^	15.46 ± 0.88 ^B^	9.45 ± 1.31 ^B^

Each value was expressed as mean ± standard deviation (n = 3). Different letters in the same column indicate statistically significant differences between samples (*p* < 0.05). FD—freeze dried sample; HAD—hot air-dried sample (at 65 °C for 48 h); FZ + HAD—samples submitted to freezing (at −20 °C for 30 days) followed by hot air drying (at 65 °C for 48 h).

**Table 8:** Impact of freeze drying, hot air drying, and hot air drying preceded by freezing on mango peel powders’ color, water absorption capacity, and oil absorption capacity.

	Color				Water Absorption Capacity g of Water/g of Sample	Oil Absorption Capacity g of Oil/g of Sample	Water Activity a_w_
	L*	a*	b*	Hue Angle			
**FD**	20.21 ± 0.46 ^A^	0.04 ± 0.00 ^C^	4.98 ± 0.16 ^C^	89.54 ± 0.01 ^A^	5.04 ± 0.07 ^B^	2.03 ± 0.11 ^A^	0.13 ± 0.00 ^A^
**HAD**	15.62 ± 0.23 ^B^	0.95 ± 0.05 ^B^	6.07 ± 0.00 ^B^	81.42 ± 0.20 ^B^	6.29 ± 0.25 ^A^	1.40 ± 0.07 ^B^	0.23 ± 0.00 ^B^
**FZ + HAD**	14.39 ± 0.41 ^C^	1.38 ± 0.15 ^A^	6.50 ± 0.11 ^A^	77.65 ± 1.15 ^C^	6.23 ± 0.37 ^A^	1.28 ± 0.15 ^B^	0.23 ± 0.00 ^B^

Each value was expressed as mean ± standard deviation (n = 3). Different letters in the same column indicate statistically significant differences between samples (*p* < 0.05). FD—freeze dried sample; HAD—hot air-dried sample (at 65 °C for 48 h); FZ + HAD—samples submitted to freezing (at −20 °C for 30 days) followed by hot air drying (at 65 °C for 48 h).

## Data Availability

The original contributions presented in this study are included in the article. Further inquiries can be directed to the corresponding author.

## Abbreviations

The following abbreviations are used in this manuscript:

FD	Freeze drying
HAD	Hot air drying
FZ + HAD	Hot air drying preceded by freezing
FS	Fresh sample
DW	Dry weight
FPC	Free phenolic compounds
BBPC	Bound phenolic compounds from basic hydrolyses
ABPC	Bound phenolic compounds from acid hydrolyses
GAEs	Gallic acid equivalents
TEs	Trolox equivalents
